# Case Report: Transverse myelitis following CAR-T cell therapy for post-transplant lymphoproliferative disorder

**DOI:** 10.3389/fimmu.2026.1794535

**Published:** 2026-04-01

**Authors:** Nicolas Desbaillets, Ana Isabel Martín-Quesada, Arthur Mulvey, Marie Le Moine, Eleonora Ghisoni, Raphael Stadelmann, Angela Koutsokera, Eleftheria Kampouri, Vincent Dunet, Andreas F. Hottinger, Petr Szturz, Caroline Arber, Lionel Trueb, Bernhard Gentner, Blanca Navarro-Rodrigo

**Affiliations:** 1 Medical Oncology, Department of Oncology, Lausanne University Hospital (CHUV) and University of Lausanne (UNIL), Lausanne, Switzerland; 2 Immuno-Oncology Service, Department of Oncology, Lausanne University Hospital (CHUV) and University of Lausanne (UNIL), Lausanne, Switzerland; 3 Ludwig Institute for Cancer Research Lausanne, Lausanne, Switzerland; 4 Service of Hematology and Central Laboratory of Hematology, Departments of Oncology and Laboratory Medicine and Pathology, Lausanne University Hospital (CHUV), Lausanne, Switzerland; 5 Pneumology Service, Pulmonary Transplant Unit, Lausanne University Hospital (CHUV) and University of Lausanne (UNIL), Lausanne, Switzerland; 6 Infectious Diseases Service, Lausanne University Hospital (CHUV) and University of Lausanne (UNIL), Lausanne, Switzerland; 7 Department of Medical Radiology, Lausanne University Hospital (CHUV) and University of Lausanne (UNIL), Lausanne, Switzerland; 8 Swiss Cancer Center Léman, Lausanne, Switzerland; 9 AGORA Cancer Research Center, Lausanne, Switzerland; 10 Lundin Family Brain Tumor Research Centre, Department of Oncology University Hospital (CHUV) and University of Lausanne (UNIL), Lausanne, Switzerland

**Keywords:** Chimeric Antigen Receptor T-cell Therapy (CAR-T cell therapy), Immune Effector Cell-associated Neurotoxicity Syndrome (ICANS), Post-Transplant Lymphoproliferative Disease (PTLD), transverse myelitis, cytokines

## Abstract

We report a case of severe, irreversible transverse myelitis following CD19-directed CAR-T cell therapy (axicabtagene ciloleucel) for refractory post-transplant lymphoproliferative disorder (PTLD) in a young female lung-transplant recipient. This case illustrates an emerging neurotoxic complication of CAR-T cell therapy and highlights convergent risk factors including demographics, tumor type and CAR construct. We discuss current evidence of CAR-T–associated myelopathy, its proposed inflammatory and immune-trafficking mechanisms, and the potential contribution of interleukins and chemokines such as IL-1, IL-6, IL-18, CCL-2 and CXCL10 signaling to spinal cord injury. We further address diagnostic challenges, and review current management strategies such as corticosteroids, IL-1 blockade, and IL-6 neutralization. This report underscores the need for heightened vigilance, early imaging, and systematic reporting to improve prevention and treatment of this rare but severe CAR-T–related neurotoxicity.

## Introduction

Post-transplant lymphoproliferative disorder (PTLD) represents a serious complication of chronic immunosuppression, occurring in 1–20% of solid organ transplant recipients depending on graft type, immunosuppressive regimen, and Epstein–Barr virus (EBV) serostatus (1). PTLD pathogenesis is largely driven by dysfunctional T-cell surveillance of EBV-infected B cells (2, 3).

Standard management involves reduction of immunosuppression and rituximab, an anti-CD20 monoclonal antibody, followed by cytotoxic chemotherapy if needed (4, 5). However, relapsed/refractory disease remains challenging, with poor survival and frequent infectious complications (1). In this setting, virus-specific T cells (VSTs), particularly EBV-directed cytotoxic T lymphocytes, represent an important immunotherapeutic approach for EBV-driven PTLD. While generally reserved for cases where standard therapies have failed or are contraindicated (6, 7), recent studies have demonstrated that third-party, partially HLA-matched VSTs can be effective and safe in both hematopoietic cell transplant (HCT) and solid organ transplant (SOT) recipients, yielding complete or partial responses even in rituximab-refractory disease (1, 8).

While CD19-directed chimeric antigen receptor (CAR)-T cell therapy has revolutionized outcomes in relapsed/refractory B-cell malignancies (9–11), evidence supporting its use in PTLD is sparse and limited to isolated case reports (12, 13). Patients with advanced relapsed PTLD (often diffuse large B-cell lymphoma (DLBCL)-type) represent a patient population with specific risks following CAR-T cell therapy, as these patients are characterized by profound immunosuppression, infection susceptibility, high tumor immunogenicity and risk of graft rejection (14).

Inflammatory toxicities following CAR-T cell therapy are frequent and manageable, including cytokine release syndrome (CRS), immune-effector cell associated neurotoxicity syndrome (ICANS), and immune effector cell-associated hematologic toxicities (ICAHT) (15), which are driven by cytokine-mediated inflammation (16–19). ICANS typically presents within 3 to 9 days post-infusion with encephalopathy (confusion, altered mental status), language impairment, dysgraphia, tremor, and fine motor impairment within severe cases, seizures or cerebral edema. Its incidence varies with population and CAR-T construct and ranges from 20–60% in adults, with severe cases in up to 30% (20). As the number of patients receiving CAR-T cell therapy rises and new products are developed, atypical neurological complications are emerging, such as Parkinson-like movement and neurocognitive disorders associated with ciltacabtagene autoleucel (cilta-cel) (21), severe ICANS with central thalamic and brainstem oedema, and rare cases of paralysis (22).

Acute spinal cord toxicity (myelopathy) is an increasingly recognized complication of CAR-T cell therapy, with early reports documenting cases of transverse myelitis in patients treated with CD19 CAR-T cells for refractory mediastinal lymphoma (23, 24). Building on these observations, a recent review systematically analyzed 24 published cases to provide a broader overview of this clinical entity. The analysis explored potential causes including severe ICANS, spinal cord infarction, on-target off-tumor toxicity, and viral infections, although the exact pathophysiology and optimal management remained largely undefined in most cases (25).

Here, we describe a case of severe, irreversible CAR-T–associated transverse myelitis in the setting of refractory EBV-positive PTLD after lung transplantation, distinguished by a fulminant course and profound immunosuppression with overlapping severe infections (COVID-19, invasive aspergillosis).

## Case report

A 24-year-old woman with a history of neonatal respiratory distress syndrome and probable obstructive bronchiolitis in the context of recurrent respiratory infections underwent bilateral lung transplantation at age 23. Her post-transplant course was complicated by acute cellular rejection requiring high doses of methylprednisolone. Three months post-transplant, she presented with an EBV-positive monomorphic PTLD (DLBCL-type), stage IV, with pulmonary and hepatic involvement.

Following four weekly doses of rituximab, the patient’s disease progressed, with new involvement of the lungs, lymph nodes, liver, kidneys, breasts, and adrenal glands. She then received one cycle of R-CHOP (rituximab, cyclophosphamide, doxorubicin, vincristine, and prednisone), which was complicated by febrile neutropenia and septic shock due to pneumonia and probable invasive aspergillosis, progressing to ARDS that required mechanical ventilation. Her recovery was further delayed by a subsequent episode of urinary sepsis. Once stable, she initiated brentuximab-vedotin, achieving a partial response, which was followed by liver progression two months later. Third-line treatment with EBV-specific T-cells (tabelecleucel) failed to elicit a response, and her course was compounded by a persistent SARS-CoV-2 infection. Due to her refractory condition, she was evaluated for CD19-targeted CAR-T treatment with axicabtagene ciloleucel (axi-cel). A bridging course of brentuximab-vedotin resulted in a dissociated response, with disease progression in the liver, confirmed by biopsy.

Given the lung transplant setting, immunosuppression required careful modulation. The patient was maintained at the time of hospitalization on a standard triple regimen of tacrolimus (2.5 mg in the morning and 3 mg in the evening), mycophenolate mofetil (250 mg twice daily), and prednisone 7.5 mg daily. In preparation for CAR-T therapy, mycophenolate was discontinued at admission (Day −5), while tacrolimus was progressively tapered with close monitoring of trough levels, aiming for complete withdrawal by the time of CAR-T infusion (Day 0). Prednisone was maintained at a low baseline dose (7.5 mg/day). Our strategy prioritized maximizing CAR-T cell expansion and antitumor efficacy, while balancing the risk of lung allograft rejection.

Per institutional guidelines, the patient received trimethoprim/sulfamethoxazole (TMP-SMX) for Pneumocystis jirovecii and toxoplasmosis prophylaxis, and valacyclovir prophylaxis for Herpes Simplex Virus (HSV) and Varicella Zoster Virus (VZV). Cytomegalovirus (CMV) prophylaxis with letermovir, initiated after lung transplant due to valganciclovir-related myelosuppression, was continued. She also remained on isavuconazole as secondary antifungal prophylaxis due to intense immunosuppression, and on remdesivir for persistent SARS-CoV-2 infection.

Lymphodepleting chemotherapy was initiated with cyclophosphamide (500 mg/m^2^) and fludarabine (30 mg/m^2^) both daily from Day −5 to Day −3. Treatment was complicated by febrile neutropenia due to respiratory infection (clinically documented infection) on Day −2, managed with cefepime and azithromycin. Imaging suggested a hepatic abscess, leading to temporary postponement of infusion for four days; however, biopsy excluded infection and confirmed necrotic PTLD involvement.

Axi-cel was infused on Day 0 (160x10^6^ CAR positive T cells). From Day +2, the patient developed a grade 1 CRS and received four doses of tocilizumab 8 mg/kg (Days +2 to +5) and anakinra (100 mg subcutaneous - s.c. every 12 hours from Day +4) for persistent and refractory fever. In this context of febrile neutropenia with persistent fever of unknown origin (no focus on CT-scan), cefepime was switched to piperacillin-tazobactam on the day of infusion, and later to meropenem plus vancomycin. On Day +5, clinical deterioration with new hypoxemia prompted escalation to dexamethasone (10 mg), followed by siltuximab 11 mg/kg, for a grade 2 CRS.

**Figure 1:**
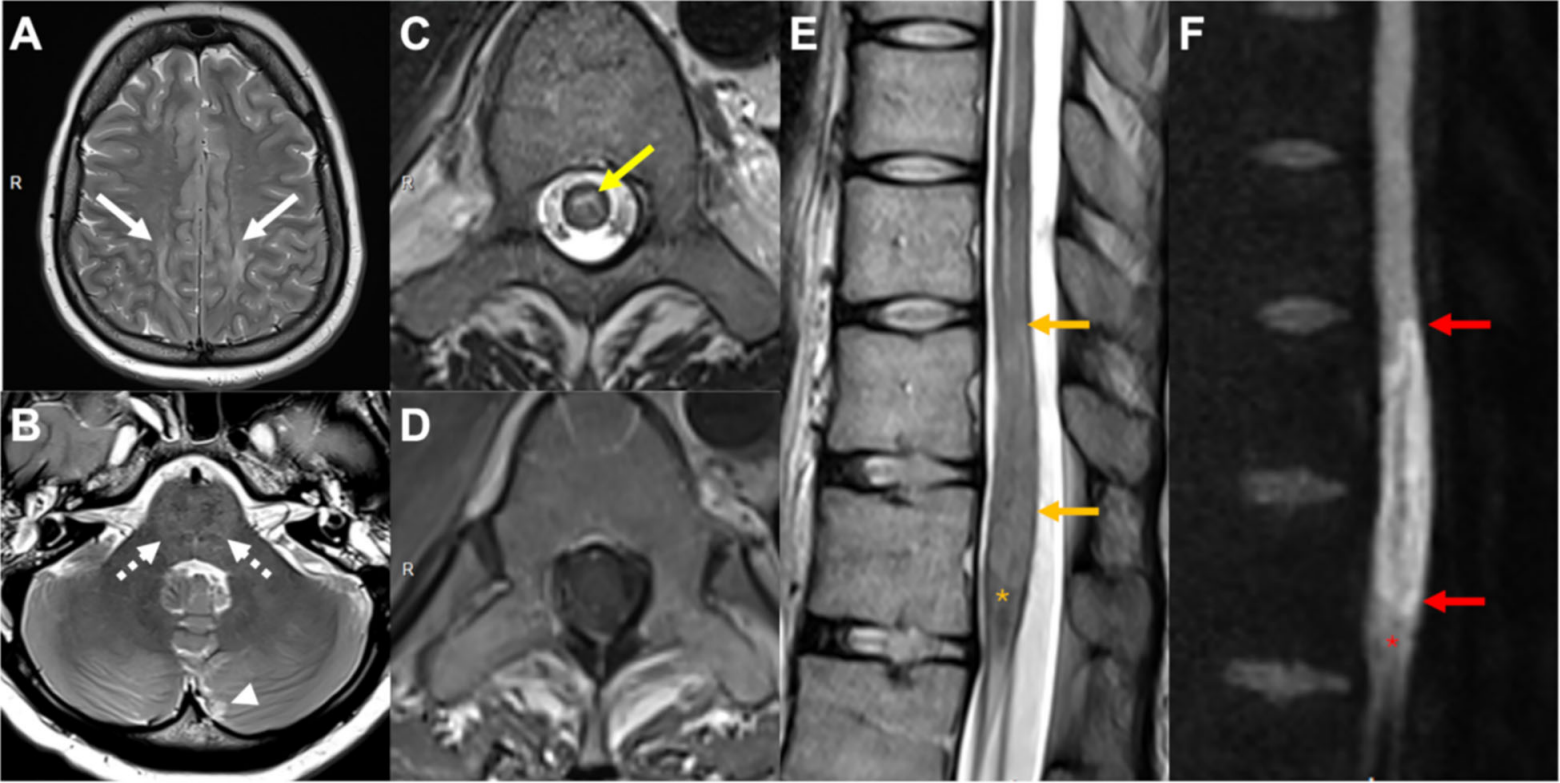
MRI findings of CAR-T–associated neurotoxicity with ICANS and transverse myelitis. **(A)** Brain MRI on Day + 5: Axial T2-weighted sequence showing diffuse supratentorial cortico-subcortical swelling (white arrows), with periventricular and deep white matter hyperintensities, and mesencephalic involvement (not shown), consistent with ICANS-related edema. **(B)** Brain MRI on Day + 5: Axial T2-weighted sequence illustrating a left paramedian cerebellar ischemic focus in the PICA territory (arrow head) that had diffusion restriction with ADC signal drop, corresponding to cytotoxic edema, as well as cortical cerebellar and brainstem swelling (dashed arrows) with high ADC values corresponding to vasogenic edema. **(C)** Spinal MRI on Day + 9: Axial T2-weighted spinal image demonstrating central gray matter hyperintensity (arrow) and edema at T11–L1. **(D)** Spinal MRI on Day + 9: Corresponding axial T1-weighted post-contrast image showing absence of enhancement, excluding infectious or neoplastic infiltration. **(E)** Spinal MRI on Day + 9: Sagittal T2-weighted sequence revealing longitudinally extensive central cord hyperintensity spanning T7–T12, including the medullary cone (star), with both grey and white matter intense swelling at T11-T12 (arrows). **(F)** Spinal MRI on Day +9: Sagittal diffusion-weighted sequence showing diffusion restriction of the spinal cord at T11-T12 sparing the medullary cone (star), which indicated the presence of cytotoxic edema at T11–12 and vasogenic edema in the rest of the cord.

On Day +5, the patient experienced an abrupt neurological deterioration with a rapid decline in Glasgow Coma Scale to 3/15 over one hour, consistent with a grade 4 ICANS associated with a concomitant grade 2 CRS. She was transferred to the intensive care unit (ICU), intubated, and treated with high-dose corticosteroids (methylprednisolone 1 g/day for 2 days and 500 mg/day 1 day), anakinra 100 mg s.c. every 6 hours, seizure prophylaxis with levetiracetam, and neuroprotective measures (mannitol, hypertension and hypothermia). Alternative causes of neurologic decline were excluded. Drug-induced neurotoxicity was unlikely: cefepime was stopped on Day 0 (a prior trough level was high-normal (17 mg/l) but asymptomatic), and therapeutic levels were confirmed for piperacillin-tazobactam, vancomycin, and isavuconazole. Oxycodone dosing was stable without renal impairment. Regarding the infectious workup, blood and urine cultures were negative. Blood PCR was negative for CMV and transiently low-positive for HHV-6 in whole blood (~300 copies/mL), with spontaneous clearance 48 hours later. SARS-CoV-2 PCR was very low positive (10³ copies/mL), while influenza and RSV were negative. Lumbar puncture was not routinely performed given the typical presentation of ICANS and thrombocytopenia. EEG ruled out seizure activity. Transcranial doppler revealed severely raised intracranial pressure. Brain magnetic resonance imaging (MRI) demonstrated diffuse edema of the midbrain, periventricular, and subcortical regions, leptomeningeal enhancement, and a new left cerebellar ischemic lesion (**Figures 1A, B**). Neurological recovery permitted extubation on Day +8.

On Day +9, following weaning of sedation, paraplegia with sensory loss below the waist was observed. Spinal MRI revealed longitudinally extensive central gray matter edema at T7–T10 sparing the white matter, as well as a large area with restricted diffusion involving the whole spinal cord (i.e. the gray and white matter) at T11–T12, but without contrast enhancement (**Figures 1C–F**). Cerebrospinal fluid (CSF) showed marked protein elevation (537 mg/L), increased lactate (2.82 mmol/L), and normal glucose. Although CSF white cell count was normal (2 x 10^6^ cells/l), flow cytometry showed 72% lymphocytosis. These were predominantly CD3+ T-cells (94%) with an inverted CD4/CD8 ratio (36/46) and 30% CAR-T cells, lacking B or NK cells. Concurrent Day +7 blood CAR levels were 13,672 copies/μg DNA. Infectious work-up of the CSF included multiplex PCR (Biofire^®^) for common pathogens and targeted PCR testing for human herpes virus 6 (HHV-6), CMV, EBV, adenovirus, JC virus and tick-borne encephalitis virus, all of which were negative. Given reports of HHV-6-associated myelitis after CAR-T cell therapy (26) this was specifically evaluated; very low-level HHV-6 DNA in blood with undetectable CSF PCR made active HHV-6 myelitis unlikely. Despite persistent detection of SARS-CoV-2 in respiratory samples, CSF PCR was negative. HIV serology and serology for hepatitis A, B and C were negative. CSF PCR for *Toxoplasma gondii* was also negative. Additional testing excluded intracellular bacteria including *Coxiella burnetti, Mycoplasma* spp*, Bartonella* spp*, Brucella* spp, as well as syphilis and Lyme disease (serology and CSF PCR). Mycobacterial infection was excluded by CSF culture and PCR. Fungal infection was excluded by culture, pan-fungal PCR, specific PCR for *Aspergillus* spp. and Mucorales, and cryptococcal antigen testing. Finally, metagenomic next-generation sequencing of CSF did not detect any pathogens. No malignant cells were detected (**Table 1**).

**Table 1:** Diagnostic work-up of CAR-T–associated transverse myelitis.

Work-up	Purpose & Details
Spinal MRI	Evaluate for compression, tumor infiltration, stroke, hemorrhage, inflammatory signs, edema
Brain MRI	Assess for hemorrhage, edema, abscess, metastatic spread
CSF analysis	Cell count/differential; protein/glucose levels; Gram stain, cultures cultures; viral PCRs (HSV-1/2, VZV, EBV, CMV, HHV-6, Enteroviruses, JC virus, HIV, HTLV-1/2, SARS-CoV-2)
ENMG	Distinguish central from peripheral involvement

Table summarizing the evaluation of the patient following acute transverse myelitis with paraplegia post CAR-T infusion.

MRI, magnetic resonance imaging; CSF, cerebrospinal fluid; PCR, polymerase chain reaction; HSV-1/2, herpes simplex virus type 1 and 2; JC virus, John Cunningham virus; VZV, varicella zoster virus; EBV, Epstein–Barr virus; CMV, cytomegalovirus; HHV-6, human herpesvirus 6; HIV, human immunodeficiency virus; HTLV-1/2, human T-cell lymphotropic virus type 1 and 2; ENMG, electroneuromyography.

Electroneuromyography (ENMG) demonstrated anterior horn cell involvement, Wallerian degeneration, and severe denervation, compatible with an immune-mediated acute transverse myelitis.

Management involved an aggressive multi-faceted immunomodulatory regimen. The patient received pulse intravenous (IV) corticosteroids, beginning with methylprednisolone 1 g/day for 2 days (Day +9 to +10), followed by 500 mg/day for 8 days (Day +11 to +18). The IV methylprednisolone was then progressively tapered until Day +28. On Day +29, therapy was switched to oral prednisone, which was initiated at 40 mg/day and tapered to 20 mg/day by Day +33.

Concurrently with the high dose steroid therapy, anakinra was continued at 100 mg every 6 hours until Day +16, at which point the dose was reduced to 100 mg every 12 hours from Day +17 to Day +20. Intravenous immunoglobulins (IVIG) were administered at 1 g/kg on Day +11 and Day +12.

**Figure 2:**
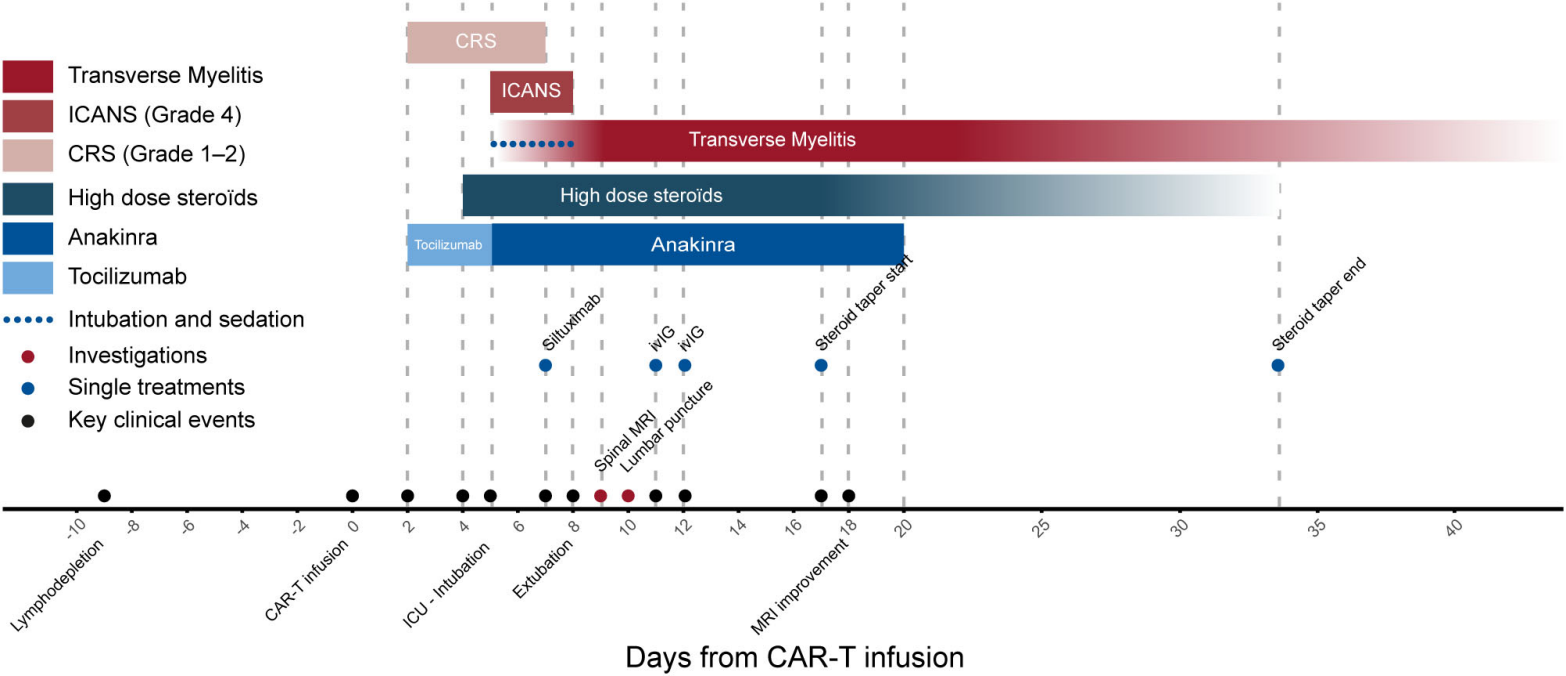
Clinical timeline of CAR-T–associated toxicities, investigations, and interventions. Timeline of clinical events and treatments following lymphodepletion and CD19-directed CAR-T infusion (Day 0). Clinical events are shown in red (bars and points), including the onset and course of CRS (Grade 1–2), ICANS (Grade 4), and transverse myelitis with paraplegia. Medical interventions are shown in blue (bars and points), including tocilizumab, anakinra, and high-dose corticosteroids, as well as diagnostic investigations such as spinal MRI, lumbar puncture, and ENMG. CRS developed on Day +2 and was managed with tocilizumab and corticosteroids; ICANS occurred on Day +5 requiring ICU admission and intubation; transverse myelitis was diagnosed on Day +9 and persisted throughout follow-up. Despite combined immunomodulatory therapies, neurological recovery remained incomplete, with radiological improvement but persistent paraplegia. CAR-T, chimeric antigen receptor-T cell therapy; CRS, cytokine release syndrome; ICANS, immune effector cell–associated neurotoxicity syndrome; MRI, magnetic resonance imaging; CSF, cerebrospinal fluid; ENMG, electroneuromyography; ICU, intensive care unit.

Despite a follow-up MRI on Day +18 showing mild regression of cerebral and spinal edema, no clinical motor recovery occurred (**Figure 2**).

As for the patient’s baseline immunosuppression for lung transplantation, tacrolimus was cautiously reintroduced on Day +13, targeting initial trough levels of 5 µg/L; the dose was later increased from Day +46 to target higher trough levels of 7–8.5 µg/L. Mycophenolate was restarted much later, at 250 mg twice daily on Day +70.

Regarding CAR-T efficacy, the initial assessment on Day +30 showed a partial metabolic response (Deauville score 4) with decreasing CAR-T transgene levels in blood (4,930 copies/µg DNA). By Day +100, the response was lost, with evidence of liver progression (Deauville 4), together with further contraction of the CAR-T transgene in blood to 1,327 copies/µg DNA.

## Discussion

Our case adds to the growing but still limited body of evidence describing acute myelopathy following CAR-T therapy. While most reported cases involve patients with relapsed/refractory B-cell lymphomas, our patient represents the unusual setting of EBV-positive PTLD in a young female lung-transplant recipient.

### Patient risk factors

In the largest published case series to date (25), most adult patients who developed CAR-T–related myelopathy were young women, often younger than the median age for DLBCL. The authors hypothesize that female patients may be predisposed to heightened immune activation due to sex-related differences in inflammatory responses and blood–brain barrier regulation (25). Our 24-year-old female patient fits this demographic profile, further supporting the possibility of a sex- and age-related susceptibility to neurotoxicity.

Published cases frequently occurred in lymphoma subtypes associated with a pre-inflamed tumor microenvironment, such as primary mediastinal B-cell lymphoma and EBV-positive DLBCL. EBV positivity may amplify immune activation and predispose to severe neuroinflammation after CAR-T therapy (25). Our case aligns with this observation, suggesting that viral-driven lymphomas, including EBV-positive PTLDs, could represent a particularly high-risk subgroup for myelopathy.

### Product risk factors

Different CAR-T cell products pose different risk of myelopathy. Most cases of CAR-T cell associated myelopathy, including this case, are described following the infusion of a CAR-T cell product using a CD28 co-stimulatory domain (28ζ CAR) (25). Expansion kinetics of CAR-T cells differ between CD28 and 4-1BB co-stimulated CAR-T cells, with a more rapid activation and cellular expansion observed with 28ζ CARs such as axi-cel. Clinically, this results in higher rates of CRS and ICANS with 28ζ compared to 4-1BBζ CARs (27). In addition, axi-cel has increased sensitivity to low CD19 antigen expression on blood-brain barrier pericytes which may contribute to a higher incidence of neurotoxicity (28).

### Clinical course

Nearly all published cases were preceded by low-grade CRS and high-grade ICANS, with myelopathy manifesting between Day +5 and +27 post-infusion. Our patient experienced grade 4 ICANS with abrupt neurological deterioration and development of transverse myelitis evidenced at Day +9, paralleling the typical pattern and supporting the concept that myelopathy may represent an extension or severe phenotype of ICANS-related inflammation.

Our patient exhibited a stepwise progression of neurological complications and spinal cord injury developed despite treatment with high-dose methylprednisolone, anakinra, and IL-6 (interleukin-6) blockade via siltuximab. This evolution suggests that the early inflammatory flare had already primed the spinal cord for injury. These findings support a ‘two-hit’ model in which axi-cel–driven inflammation and endothelial activation disrupt the blood–spinal cord barrier, enabling CAR-T cells trafficking into the central nervous system (CNS) (25, 29). MRI-detected edema, CSF detection of CAR-T cells, and ENMG-confirmed anterior horn involvement collectively suggest direct CAR-T–mediated myelopathy, a mechanism further supported by cases of leukoencephalomyelopathy with CSF CAR-T and myeloid cell infiltration reported (29).

### Cytokine dynamics

Following CAR-T infusion, our patient exhibited a robust systemic inflammatory response. A sharp IL-6 peak (Days +2 to +5) coincided with CRS and early ICANS, curtailing post-siltuximab. Concurrently, chemokines reflecting systemic macrophage and microglia recruitment (CCL2, CXCL10, CXCL9) (30–32) elevated early and subsequently declined upon the scalation of steroids (33–35). Systemic IL-18 remained persistently elevated, indicating a refractory hyperinflammatory state (36–38) that subsided only after high-dose methylprednisolone (37, 38).

Interestingly, Interferon gamma (IFN-γ) and tumor necrosis factor-alpha (TNF-α) remained low, diverging from the cytokine fingerprints typically seen in published CAR-T recipients’ series (39, 40). This muted profile may reflect chronic immunosuppression and prior viral infections altering immune responses.

**Figure 3:**
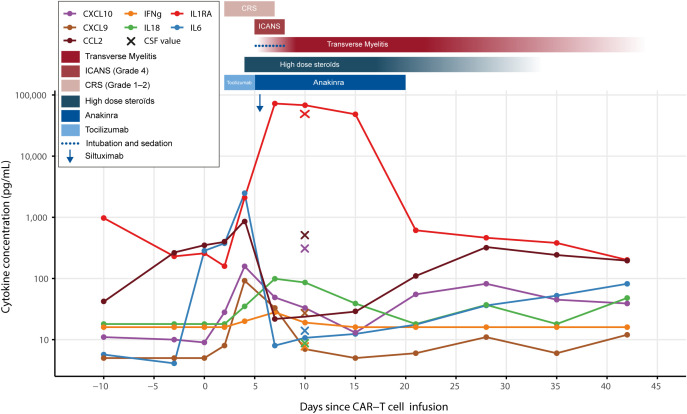
Cytokine dynamics in relation to CAR-T–associated toxicities. Longitudinal serum cytokine concentrations (logarithmic scale) following CAR-T infusion (Day 0). IL-6 (blue) peaked sharply between Day +2 and +5, coinciding with the onset of CRS (Grade 1–2), early ICANS (Grade 4) and tocilizumab administration; levels decreased on Day +6 after siltuximab infusion. IL-1RA (red) rose early and peaked during anakinra treatment. CCL2 (burgundy), CXCL10 (purple), CXCL9 (brown) increased until Day +5 then declined to baseline levels following the initiation of high-dose corticosteroids and anakinra. IL-18 (green) demonstrated fluctuating elevations, while IFN-γ (orange) showed moderate early increases that declined thereafter. CSF cytokines (depicted by crosses) followed a similar trend to serum cytokines, with the exception of CCL2 and CXCL10, which were markedly elevated, and IL-18, which remained low. Clinical toxicities are represented by red bars (CRS, ICANS, transverse myelitis), whereas therapeutic interventions are shown as blue bars (tocilizumab, anakinra, high-dose corticosteroids), with siltuximab administration marked by the downward arrow. CAR-T, chimeric antigen receptor T-cell therapy; CRS, cytokine release syndrome; ICANS, immune effector cell–associated neurotoxicity syndrome; IL, interleukin; IFN-γ, interferon gamma; CNS, central nervous system.

CSF analysis at transverse myelitis onset, revealed a particular compartmentalization. While IL-18 was very low in the CSF—suggesting a primarily systemic role—CCL2 and CXCL10 were markedly enriched compared to concurrent serum levels. This gradient may reflect local intrathecal production and active CNS immune trafficking rather than passive diffusion. These findings align with established literature demonstrating that CSF enrichment of CXCL10 and CCL2 is a hallmark of severe ICANS (41, 42). **Figure 3** depicts the cytokine dynamics in relation to the acute clinical manifestations and the underlying immune cascade.

### Management

From a diagnosis perspective, these trajectories emphasize the need for increased clinical awareness and early recognition especially in presence of risk factors (young, female, EBV positive or pre-inflamed tumor microenvironment). Atypical or progressive neurological deficits, even as systemic parameters appear to normalize should trigger a dedicated work-up. Moreover, reliance on systemic cytokine monitoring alone may underestimate ongoing CNS injury, highlighting the importance of CSF analysis and spinal imaging.

Differential diagnoses included lymphoma progression and spinal cord infarction secondary to elevated cerebrospinal fluid pressure, as described previously (25). However, the pattern of transverse spinal involvement, unrelated to vascular territories, and the atypical MRI features for lymphoma progression or an ischemic lesion made these alternatives unlikely.

Importantly, diagnostic work-up should exclude ischemic, hemorrhagic, metabolic and infectious causes. HHV-6 encephalitis can occur after CAR-T cell therapy, and HHV-6 myelitis in particular has been reported (26). Because manifestations can be clinically indistinguishable from ICANS, HHV-6 should be considered in patients with neurological symptoms not resolving with ICANS management. In such cases, PCR testing in blood and CSF is essential (26, 43). HHV6 infection has been actively excluded in our patient.

CAR-T cell–mediated myelitis is mainly managed according to ICANS consensus (44, 45); although recently reviewed, its treatment remains largely empirical (25). Tocilizumab, with poor CNS penetration and potential to elevate CSF IL-6, should be reserved for concurrent CRS, while prompt high-dose corticosteroids remain the mainstay, with consideration of IL-1 blockade (e.g., anakinra) in severe or refractory cases (25). In selected cases, IVIG and plasma exchange provide limited benefit (17).

Very few reported patients achieved full neurological recovery, underscoring the risk of irreversible axonal injury. IL-6 appears pivotal in the early inflammatory cascade that precipitates CAR-T–associated myelopathy, which is frequently followed by persistent neurological deficits. The limited efficacy of IL-6 blockade in this context further distinguishes its pathophysiology from classical CRS. Retrospectively, IL-6 levels of our patient were increased prior to CAR-T cell infusion. Elevated pre-infusion IL-6 levels positively correlate with higher grade CRS and higher post-infusional IL-6 peak values upon CAR-T treatment (46). Emerging evidence suggests that direct IL-6 neutralization with siltuximab, unlike IL-6 receptor blockade, may attenuate both CRS and ICANS by reducing circulating and cerebrospinal IL-6 levels (24, 47). Case reports have shown rapid neurological improvement and decreased CSF IL-6 following siltuximab administration, even in CAR-T–related myelopathy, supporting its ability to cross the disrupted blood–brain barrier during neurotoxicity episodes (48). IL-1, an upstream driver of myeloid activation and IL-6 release, contributes to early cytokine amplification (49); blocking IL-1 receptor signaling with anakinra, the recombinant IL-1 receptor antagonist with excellent CNS penetration, has shown encouraging results in steroid-refractory ICANS and CRS, where earlier and higher-dose administration (>200 mg/day) was associated with reduced mortality and lower neurotoxicity rates (50). Prophylactic or early anakinra initiation in high-risk CAR-T recipients markedly reduced both all-grade and severe ICANS without impairing anti-tumor efficacy in recent phase II trials (17, 50).

Our patient started anakinra treatment on day 4; whether earlier treatment with anakinra and siltuximab would have changed the clinical course is unknown. Nevertheless, timely recognition and early spinal imaging are essential to guide intervention and potentially mitigate long-term disability.

## Conclusion

This case illustrates a rare but serious complication of CD19-directed CAR-T cell therapy: acute, irreversible transverse myelitis, in the setting of EBV-positive PTLD after lung transplantation. Our case mirrors several recurrent features described in the literature: young female gender, EBV-driven lymphoma, CD28 CAR construct, severe ICANS, and a systemic hyperinflammatory state. These converging risk factors may have predisposed to severe neurotoxicity and myelopathy. Despite intensive multimodal immunosuppression with corticosteroids, siltuximab, anakinra, and IVIG, the neurological deficit persisted, underscoring the poor functional prognosis often associated with this complication. Early recognition, dedicated neurological work-up, and tailored interventions such as anakinra prophylaxis in the presence of risk factors and rapid administration of corticosteroids are essential. Systematic reporting is needed to improve understanding, prevention, and management of this emerging syndrome.

## Patient perspective

The patient described her hospitalization as an intensely difficult and traumatic period, particularly her stay in the intensive care unit following Grade 4 ICANS, the diagnosis of transverse myelitis, and its sequelae. Specifically, the permanent paraplegia and the necessity of a permanent urinary catheter have been a profound psychological burden. She acknowledged that adjusting to life in a wheelchair and the loss of physical independence was emotionally exhausting during the prolonged hospitalization. However, despite the devastating functional outcome, she expressed gratitude for the supportive environment and the compassionate care she received from the medical team throughout this transition.

## Data Availability

The data presented in this article is not readily available because it contains information that could compromise the privacy of the research participant. Requests to access the datasets should be directed to blanca.navarro-rodrigo@chuv.ch.
